# Changes in Volunteering of Older Adults in the Time of the COVID-19 Pandemic: The Role of Motivations

**DOI:** 10.3390/ijerph192214755

**Published:** 2022-11-10

**Authors:** Andrea Principi, Davide Lucantoni, Sabrina Quattrini, Mirko Di Rosa, Marco Socci

**Affiliations:** 1 Centre for Socio-Economic Research on Ageing, IRCCS INRCA—National Institute of Health and Science on Ageing, 60124 Ancona, Italy; 2 Unit of Geriatric Pharmacoepidemiology and Biostatistics, IRCCS INRCA—National Institute of Health and Science on Ageing, 60124 Ancona, Italy

**Keywords:** older volunteers, active ageing, Volunteer Functions Inventory, emergency situations, Italy

## Abstract

This paper elucidates the relationship between possible changes in volunteering experienced by older people during the COVID-19 pandemic, and their motivation to volunteer, as well as the direct or indirect experience of COVID-19 symptoms. Given the well-known positive benefits of volunteering in older age both for individuals (in terms of improved health and wellbeing) and society at large, there is a paucity of studies on older volunteers in the time of COVID-19. In this context, older people’s volunteering was highly challenged due to age-based physical and social restrictions put in place by national governments, which have been considered as ageist by a large part of the gerontological scientific community. This study was carried out on a sample of 240 Italian older volunteers. The results suggest that during the COVID-19 pandemic, especially older volunteers driven by social goals (e.g., opportunities to have relationships with others) were able to continue volunteer activities without needing to change them. The study also clarified that having directly or indirectly experienced COVID-19 symptoms did not influence changes in voluntary activities of older people. These results have important policy implications, given the indication that through volunteering, older individuals may try to counter the undesired calls by the governments for self-isolation and physical distancing. It is important that in emergency situations involving older people, policy makers should not treat them as only recipients of health and social care, but also as useful providers of help in the community.

## 1. Introduction

In an era characterized by a trend of population ageing, the scientific literature ascertained that volunteering by older people is linked to benefits both for older individuals and for society as a whole [1,2]. Among the many benefits at the individual level, previous studies found that volunteering increases mental, social and physical wellbeing [3,4,5,6]. Furthermore, society at large can count on a meaningful productive input of older volunteers. For instance, in the US, their contribution has been annually estimated at over 73 billion dollars [7], and older volunteers report more median annual volunteer hours than younger ones [2].

The voluntary sector played many important roles in facing the emergence of COVID-19 [2]; however, the need for more volunteers to support individuals affected by the virus or to assist in the delivery of essential activities was evident [8]. Focusing on older volunteers, their role was highly challenged during the pandemic. Indeed, based on the assumption that older people are more vulnerable to the virus than younger ones [9,10], many forms of age-based restrictions were introduced worldwide by national governments. In some cases, people aged 70 years or more were asked to self-isolate [11]. In most cases, older people were excluded from in-person societal activities [12], as it was the first group of individuals encouraged or advised to stay home [13,14] and to cease physical contact with children and grandchildren [15]. All this led to inevitable social isolation [16,17].

These restrictions were reasonably motivated by the fact that older people, and especially those with chronic diseases, manifested much higher infection and mortality rates of COVID-19 [18]. On the other hand—and both public announcements and social media did not help in this respect [19]—the restrictions did not take into consideration that older people could not be treated as a homogeneous group since they differ in life experiences, cultural backgrounds, genetics and health histories [15,19,20]. They also have different abilities, preferences and motivations [2]. Thus, being subject to the “tyranny of averages” [11] which neglects older people’s diversity, this approach was considered as discriminatory and facilitated an increase of already existing inequalities [21].

The ageist implications of the “chronological quarantine” have been emphasized in many countries as evidence of unprecedented state intervention of extraordinary severity [11,22,23,24,25,26]. Age-stereotyping individuals, in this respect, was deemed by many to be a sign of unawareness regarding the value that should be attributed to older people [13]. The prevalent view became that of considering older people as helpless, frail and unable to contribute to society [19], denying the importance of their involvement in social and economic life [27] as well as their right to assess their own risks and make autonomous decisions, regardless of the circumstances [17]. Since social contribution and opportunities are self-protective actions which produce a sense of purpose and drive, the interruption of social actions, including volunteering, threatened older people [2,28].

Ageist practices observed during the pandemic caused practical negative consequences for older people, such as lower subjective health, wellbeing, quality of life and life satisfaction [1,15], as well as due to disruption in usual services. Negative effects were also related to the emotional sphere, through increased depression and anxiety symptoms [13,16].

Evidence concerning the difficulties encountered by older people in accessing volunteering during the pandemic was largely available. Despite the fact that volunteering is an important activity in the lives of many older adults, volunteer opportunities had been highly reduced or had disappeared [29]. For instance, older volunteer drivers could no longer fulfil their assignments [13], thus jeopardizing their important contributions to the crisis response [30]. Grotz and colleagues [31] observed that the sudden cessation of volunteering during the COVID-19 pandemic caused negative health and wellbeing effects for older volunteers.

However, a certain number of older people managed to volunteer during the crisis. Volunteer experiences in this case concerned, to a considerable extent, retired older adults with a professional background in the medical field (e.g., nursing, medicine and social work) [2], who were recruited to volunteer to help fight the health crisis. This, to some extent, also concerned older people helping online with home schooling [32,33] in the context of increasing interest and attitudes of older adults toward virtual volunteering during the pandemic [34]. Interestingly, rather than COVID-19 acting as a discouraging factor, a study found that the willingness to be useful during the pandemic may have helped to engage older people who would not usually volunteer [8].

The limited number of older volunteers who had managed to continue volunteering during the health crisis experienced benefits such as fewer depressive and anxiety symptoms and lower levels of anger and frustration [16,35]. In light of this, the encouragement of volunteering among older people can be considered as a useful strategy in order to maintain their mental health during emergency situations such as the COVID-19 outbreak [16]. The fact that this strategy has not properly been planned and concretely supported with adequate measures can be considered to be a lack of preparedness at the policy making level, particularly regarding the inability to consider the societal impact of the COVID-19 outbreak beyond the immediate medical emergency response [31].

From the above observations, it is clear that even though volunteering may be considered as a health and wellbeing protective factor for older adults, even during a pandemic, such a pandemic causes volunteer opportunities for them to be highly reduced. However, empirically speaking, changes in volunteering attitudes of older people during the COVID-19 outbreak were scarcely investigated, and for this reason, to date, there is extremely limited evidence on the subject.

For example, a UK study based on ELSA data focused on volunteering in older age through a sub-study concerning changes, and found that 61% of older volunteers were more likely to have decreased (18%) or stopped (43%) volunteering, while only 9% increased their involvement. Older workers were less likely to stop volunteering than non-working people, while being wealthier was associated with decreasing volunteer engagement. Older people having functional difficulties and those reporting COVID-19 symptoms were less likely to increase volunteer activities [36]. In the same country, another study conducted on people aged 70+, which focused on volunteering after the COVID-19 outbreak, found that 17% of older people continued with formal volunteering, 9% stopped their engagement in voluntary work and 6% started volunteering [37].

A further UK study carried out on a sample of volunteers of all ages (18+), found that 23% decreased volunteering during the pandemic compared to their prior involvement, while 12% increased their engagement and the rest reported about the same amount. Associations with decreased volunteering were found with being divorced, being of poorer physical health and being neurotic [8].

In light of the scarce evidence available on the matter [16], this paper will contribute to the literature by studying changes in the volunteering habits of older people during the pandemic, in the Italian context.

### 1.1. The Italian Context during the Pandemic

In Italy, COVID-19-related containment measures have had a cyclical cadence, in line with the various pandemic waves. The periods of lesser intensity of the emergency were those between June and September, both in 2020 and 2021.

Regarding the main measures undertaken by the Italian government to combat the health crisis, on 31 January 2020, a state of emergency on the national territory was declared for six months [38], and then periodically renewed until 31 March 2022. From 9 March to 3 May 2020, a lock-down across the entire national territory was established, during which, it was prohibited to go out of one’s house except for reasons of work, health or to purchase primary goods [39]. This phase was characterized by rules of physical distancing and had the objective of reducing the pressure on the intensive care units in the hospitals. Later, from May to June 2020 [40], since the epidemic curve was in a downward phase, a gradual easing of the previous containment measures was implemented, to the point that various productive activities had reopened. Then, from July to October 2020, regional governments could manage their own re-opening measures, based on the epidemiological situation of the territories [41]. From October 2020 to April 2021, following the rise of the contagion curve at a regional level, a national classification system based on different zones (yellow, orange and red) was instituted in order to allow different grades of restrictive measures depending on the severity of the health emergency in each region, together with a national mandatory curfew from 10:00 pm to 5:00 am [42,43]. In the yellow area, it was mandatory to wear a mask outdoors. Schools, shops, bars and restaurants were open without time limits. In the orange area, shops, bars and restaurants were open with time and service restrictions. From both orange and red regions, it was not possible to move into other regions. In red areas, individuals were not allowed to move, even within one’s own municipality (except for work or health reasons); non-essential shops, restaurants and bars were closed, as well as were schools (distance learning tools were enabled). With the subsequent improvement of the situation, in light of the encouraging scientific data on the epidemic and the progress of the vaccination campaign, a progressive elimination of the restrictions took place [44], and all the Italian regions were classified as “white areas” for the period between June and December 2021 [45], with a stated obligation to wear masks indoors; all activities were open and there were no restrictions on movement among regions.

During the period between July and August 2021, when the present study was conducted, the nationwide incidence showed an increase in diagnosed COVID-19 cases due to the loosening of mitigation measures, as well as the simultaneous diffusion of the delta variant, which is characterized by a greater transmissibility. Within this context, the median age of subjects who contracted the infection in this period was, however, very low (age 26–32), while only 9.1% of infected individuals were aged 60 or over [46].

### 1.2. Aims of the Study

Several studies demonstrated that there are various types of motivations for which individuals volunteer. People volunteer due to needs and motives important to them [47], thus, volunteering reflects different underlying processes [48]. Studies concerning motivational factors of older people related to volunteering are largely available [48,49,50,51,52,53]. However, there is scarce evidence regarding the volunteer motivations of older volunteers that were active during the health crisis, and in particular, their relationship with changes in volunteer activity. Due to the described age-related restrictions, older adults encountered many difficulties in performing volunteer activities during the pandemic. In this context, we hypothesize that the motivations to volunteer played an important role regarding their decisions about changes, in terms of the activities carried out and time invested in volunteering. We assume that especially more motivated older volunteers were able to guarantee a certain continuity compared to the volunteering that was carried out in the pre-COVID 19 era. For this reason, in order to fill a gap in knowledge [8], an aim of this study is to explore whether the motivations to volunteer had a role in contributing to changes in volunteering by older people during the pandemic.

A further aim of the study is to understand whether having had an experience (both direct or closely indirect, i.e., of family members or fellow volunteers) with infection by COVID-19 may have had an influence on the decision whether or not to change the volunteer experience. It may be hypothesized that experiencing the infection, either directly or indirectly, discourages the normal execution of social activities, and thus, volunteer activities as well.

In light of the above, this study poses two main research questions:

1. Do volunteer motivations play a role in determining changes to the volunteering service provided by older people in the time of COVID-19? (RQ1);
2. Does the close (direct or indirect) experience of a contagion of COVID-19 affect changes in the volunteering service provided by older people? (RQ2).
3. The results of this study add to the literature by pointing in the direction that the older population is heterogeneous, so it is necessary to avoid having one-size-fits-all policies for older adults. They demonstrate that the ageist approaches adopted by governments during the COVID-19 pandemic are inappropriate, and that when faced with possible future emergency situations, older people should not be treated as a homogeneous group. Additionally, by exploring the motivations behind older people’s volunteering habits, the results are of value to voluntary organizations’ managers who deal with recruiting and sustaining volunteers.

## 2. Materials and Methods

### 2.1. Sampling

This exploratory study was conducted between July and August 2021 in the Marche region (Central Italy) on a sample of older volunteers (aged 55 years or more) of the regional branches of the three larger voluntary organizations of older people in Italy: AUSER (Self-management of solidarity services—Association for active ageing); ANTEAS (National association of all ages and solidarity) and ADA (Association for the rights of older people). A cluster-sampling method was adopted, using each single volunteer organization as a sampling unit. The researchers conducting the study contacted regional presidents of the mentioned organizations, asking them to invite older people volunteering in these organizations to fill out an online questionnaire, prepared through LimeSurvey, articulated in the following sections: socio-demographic; volunteering activities carried out; the impact of the COVID-19 outbreak on personal circumstances and on various aspects of volunteering in older age. The final sample consisted of 240 older volunteers.

### 2.2. Measures

#### 2.2.1. Dependent Variables

Possible changes in volunteering during the pandemic have been measured by asking older volunteers: “During the pandemic…” (answer categories Y/N): (a) I continued to carry out the same volunteer activities; (b) I changed volunteer activities; (c) I increased volunteering time; (d) I decreased volunteering time.

#### 2.2.2. Independent Variables

Volunteer motivations were measured through the Volunteer Functional Inventory (VFI). The VFI is an instrument designed by Clary and colleagues [47] to measure motivational functions to volunteer in the following six domains: values (related to altruistic beliefs); understanding (learning new skills and exercising knowledge and abilities through volunteering); social (volunteering as an opportunity to have relationships with others and to conform to normative influences); career (career-related benefits from volunteering); protective (volunteering in order to protect the ego from negative problems) and enhancement (desire for personal growth and development through volunteering). This tool has been extensively applied to study volunteer motivations of older people internationally [54,55]. The VFI has also been employed in Italy to study: country differences regarding older volunteers’ motivations [48]; the relation of work status to volunteer motivations [53] and motivations to volunteer related to individual resources [52]. In the present study, for the first time, this tool was applied to understand the possible influences of volunteer motivations on changes in the volunteering habits of older adults in the time of COVID-19. On the VFI, respondents indicated the importance of each one of 30 items (each function was covered by 5 items) on a five-point Likert scale ranging from not important (1) to very important (5). Examples of questions measuring the different motivational functions are the following: I feel it is important to help others (values); volunteering lets me learn things through direct hands-on experience (understanding); volunteering increases my self-esteem (enhancement); people I know share an interest in community service (social); volunteering helps me work through my own personal problems (protective); I can make new contacts that might help my business or career (career).

To measure the direct or indirect experience of infection by COVID-19, the following question was asked: “Did the pandemic have an impact on your health or on the health of somebody you know?” Answer categories: (a) I have had COVID-19; (b) a family member has had COVID-19; (c) an acquaintance (including fellow volunteer) has had COVID-19.

#### 2.2.3. Co-Variates

As control variables, we included age; gender; marital status (married/cohabiting; single/divorced/separated; widowed); educational level based on the International Standard Classification of Education (ISCED 0: Early childhood education (‘less than primary’ for educational attainment); ISCED 1: Primary education; ISCED 2: Lower secondary education; ISCED 3: Upper secondary education; ISCED 4: Post-secondary non-tertiary education; ISCED 5: Short-cycle tertiary education; ISCED 6: Bachelor’s or equivalent level; ISCED 7: Master’s or equivalent level; ISCED 8: Doctoral or equivalent level), grouping levels 0 to 2 as “low education”, 3–4 as “intermediate” and 5+ as “high”; informal family elder care (Y/N) and volunteering frequency (twice a week or more often; once a week; once a fortnight; less than once a fortnight). It is not an aim of the paper to discuss the control variables in great detail.

### 2.3. Sample Description

The description of the sample is provided in Table 1. The mean age of the study participants is a bit over 70 years, with a slight prevalence of men over women. The large majority of the sample is married/cohabiting, while 16.3% and 12.1%, respectively, are single/separated/divorced or widowed. Concerning the educational level, most representative categories are both the intermediate level (44.2%) and the low level (38.3%), while older people with a high level of education were the less represented (17.5%).

In line with previous literature (e.g., [52,56,57,58]), across the six VFI factors, the altruistic motivational drive was the most important in the sample, while career-related motivations had the lowest score. 13% of the investigated older volunteers had direct experience with COVID-19 by getting sick themselves, while higher percentages were found with an indirect experience of the illness: 28% reported that a family member experienced COVID-19 and 46.5% reported that an acquaintance experienced COVID-19.

Changes in volunteer activities concerned 61.1% of the sample, and this percentage was higher compared to that of older volunteers who continued to carry out the same activities as before the pandemic (49.8%). In relation to volunteering time, 41% of the sample decreased it due to the COVID-19 outbreak, while those who increased time devoted to volunteering during the pandemic were 23.1%.

Most of the sample (51.3%) was made of very committed volunteers, in terms of volunteering frequency (i.e., twice a week or more often). Lastly, 12.9% of the sample was also involved, in parallel to volunteering, in informal family elder care activities.

### 2.4. Statistical Analyses

Continuous variables were reported as mean and standard deviation while categorical variables were expressed as absolute frequency and percentage. In bivariate analyses, volunteering motivational functions were compared between volunteering-related outcome variables by Student’s *t*-test, while relationships between COVID-19-related factors and volunteering-related outcome variables were assessed by Pearson’s Chi-square test.

In order to control for potential bias and confounding effects, four multivariable logistic regression models were estimated for each volunteering-related outcome variable. In Model 1, unadjusted estimates of the association between motivational factors and study outcomes were reported; in Model 2, unadjusted estimates of the association between COVID-19-related factors and study outcomes were reported. In the third model, the independent variables employed in Model 1 and Model 2 were combined (Model 3). In the last model, the independent variables employed in Model 3 were adjusted for other control variables (Model 4). For logistic models, Odds Rations (OR) were reported. A 2-tailed *p* value < 0.05 was considered significant. Data were analyzed using STATA version 15.1 (StataCorp, College Station, TX, USA).

## 3. Results

Table 2 describes the relationship between motivational factors and the dependent variables used to study changes in volunteering.

Increased volunteering time shows a significant relationship with all six motivational factors. In each of these six cases, older volunteers who increased their time devoted to volunteering had a higher motivational score compared with older volunteers who did not increase their time devoted to volunteering.

Decreased volunteering time showed a significant relationship with three motivational functions, namely social, protective and career-related motivations. In this case, older volunteers who decreased their time devoted to volunteering had a lower motivational score compared with older volunteers who did not decrease their volunteering time.

Additionally, the same three motivational factors just mentioned had a significant connection with the subjects having continued to carry out the same volunteering activities as in the pre-pandemic era. In each of these three cases, older volunteers who continued to carry out volunteer activities as before showed a higher motivational score than older volunteers who did not.

A status of having changed volunteer activities due to the pandemic did not have a significant association with any of the six motivational factors.

Table 3 shows bivariate analyses about the potential association of possible changes in volunteering and having had a direct or indirect experience with COVID-19.

This association does not seem to be very strong, since across the 12 relationships tested, in only one case were significant results obtained; that is, having been able to continue to carry out the same volunteer activities was associated with acquaintances who got sick due to the pandemic.

In order to answer the research questions of this study and to go beyond the results obtained through bivariate analyses, by employing incremental models, we regressed the four dependent variables describing possible changes in volunteering during the COVID-19 pandemic, controlling for the influence of the independent and other control variables.

Table 4 shows the results regarding the relationship of the dependent and control variables on having continued to carry out the same volunteer activities as before the pandemic era.

Across motivational functions, among the three out of six functions which resulted as significant in bivariate analyses (social, protective and career), the social motivational function has maintained the most significant level across models, in which, the greater this kind of motivation, the greater the probability of carrying out the same volunteer activities as before the pandemic era. More precisely, an increase of one point in the social motivational function is associated with an increase of 2.43 points in the probability of continuing to carry out the same volunteering activities (see Model 4).

As for direct or indirect experience with COVID-19, while in bivariate analyses, acquaintances which experienced COVID-19 had a significant role, this significance was confirmed in Models 2 and 3, but not in Model 4, when control variables were also introduced. Among the latter, the frequency of volunteering resulted as important, with more committed volunteers (those volunteering twice a week or more often) with higher probability of carrying out the same activities as before the COVID-19 outbreak.

In Table 5, the results of the regressions on changes to volunteering activities are shown.

Confirming the results obtained in bivariate analyses in this respect, none of the independent variables are associated with changes in volunteer activities. In Model 1, social-related motivations resulted as significant, but the significance disappeared in Models 3 and 4. Out of the control variables considered in the study, again, volunteering frequency played a role. In this case, older volunteers who volunteered less frequently (i.e., once a week or every two weeks) had a higher probability of changing volunteer activities. A phenomenon, the latter, was also explained by being widowed (as compared with being married).

In Table 6, the four Models are applied to the increase of volunteering time as an outcome variable.

Although, in bivariate analyses, all six motivational functions were significantly associated to this outcome when individually tested, after running Models 1 and 3, only the altruistic-related motivational factor (values) kept a level of significance, which disappeared in Model 4, with the complete set of variables included. In Model 4, increased volunteering time during the pandemic was only associated with volunteering more often (twice a week or more often) as compared with volunteering less often (i.e., once every two weeks).

Table 7 reports the results concerning explanatory variables for decreasing volunteer time due to the COVID-19 pandemic.

While in bivariate analyses, three motivational functions resulted as statistically significant (social, protective and career) in association with the reduction of volunteer time, only social-related motivations maintained a certain role in the regressions (i.e., the more this motivation, the less the reduction of time), which reached the level of statistical significance in Model 3, while it resulted only close to significance in the others. Again, volunteering frequency played a role, with the results in this respect showing that the lower the frequency (once a month or less often), the greater the possibility of decreasing volunteering time. Additionally, widowed older volunteers may be subject to a reduction of volunteering time in comparison to married/cohabiting older volunteers.

## 4. Discussion

Most of the European governments’ decisions regarding older people in relation to the COVID-19 pandemic created a short-circuiting situation around their health and wellbeing. Especially in the first pandemic waves, older people were forced to self-isolate at home in order to guarantee safer and healthier lives. However, this, on the one hand, negatively affected older people by promoting depression, anxiety and frustration [16,35], and on the other hand, made it harder for older volunteers to continue carrying out volunteer activities, which, in turn, prevented them from enjoying the many physical and mental health benefits of volunteering, and the benefits of wellbeing in general [16].

Against this background, and in light of the substantial lack of knowledge in this respect [8], this study investigated possible changes in the volunteering habits of older people during the pandemic, in terms of activities carried out and time devoted. In particular, the main interest of this study was to understand the existing relationship between changes in volunteering and: (a) volunteers’ motivations; (b) having had a direct or indirect experience with COVID-19 symptoms.

RQ1 was concerned with the role of volunteer motivations. The general hypothesis of the study was that especially more motivated older volunteers would have been able to guarantee a certain continuity in volunteering, compared with volunteering carried out before the COVID-19 outbreak. Interestingly, we found that this was true, especially for social-related motivations. This may mean that by continuing volunteer activities, volunteering becomes an important tool used by older volunteers to meet their needs for social relationships, and to counter the restrictions in this respect imposed by the government. Another motivational function worth mentioning is the altruistic one (values), which had a certain role (significant in Models 1 and 3 and close to it in Model 4) in relation to their decisions to increase volunteering time. It is consistent, indeed, that especially those older volunteers pulled by the need to help others wanted to commit more in emergency times. Interestingly, within cross-country studies conducted in the pre-COVID-19 era [48,53], volunteer motivational functions associated with Italian older volunteers were also “understanding” (desire to learn new things), “enhancement” (desire for personal growth) and “protection” (desire to protect the ego from negative feelings), which however, put now in relation with possible changes in volunteering during the pandemic, did not emerge as particularly important elements.

In general, the present study seems to indicate that the volunteering habits of older people in Italy may have been more affected by the pandemic than the volunteering habits of older people in England [36]. This, both in terms of increased (23.1% versus 9% reported in the study by Chatzi and colleagues) [36] and decreased (41% versus 18%) volunteering times. Referring to the general situation, rather than specifically to the direct or indirect experience of COVID-19, in the UK, it was found that this emergency situation acted as an encouraging, rather than a discouraging factor to volunteering, due to the willingness to feel useful during the pandemic [8]. This is also linked to the altruistic-type of volunteer motivations, that, as mentioned above, in this study, were linked, to a certain extent, to a possible increase of volunteering time.

RQ2 focused on the possible role of a direct or (closely) indirect experience with COVID-19 symptoms. In this respect, in line with evidence from the UK regarding the lesser likelihood of an increase in volunteer activities linked to reporting COVID-19 symptoms [36], the general hypothesis of this study was that the fear generated by this (direct or close) experience, would have discouraged the normal execution of volunteer activities. The latter hypothesis has not been supported. Indeed, both cases of older volunteers or their family members having experienced COVID-related symptoms did not result as significant factors in relation to possible changes in volunteering. The only input in this respect concerns COVID-19 symptoms experienced by an acquaintance in relation to being able to continue to carry out the same volunteer activities. However, in line with the results by Mak and Fancourt [8], in this case, this appeared to be an encouraging rather than a discouraging factor. However, above all, and most importantly, this association disappeared when the full model (Model 4) was run. The general message from this result, is that experience with COVID-19 symptoms did not discourage older people from carrying out their usual volunteer activities.

Across all control variables included in the analyses, volunteering frequency and marital status were shown to play a role in the volunteering habits of older people in the COVID-19 era. Concerning volunteering frequency, the general result is that, as opposed to older volunteers who volunteer less frequently, older volunteers who volunteer more often (twice or more a week) seemed to have been able to guarantee the execution of their volunteer activities and to increase, rather than to decrease, their volunteering time. As for marital status, this study indicates that during the pandemic, volunteer activities may have been especially jeopardized for widowed older people who had to adapt their volunteer activities by also reducing their volunteering time. This may also be linked to the higher age of widowed older volunteers, even if, in this study, age never emerged as a significant factor. The results of this study, in this respect, are partially different than those obtained by Mak and Fancourt [8] in the UK, since, while exploring a sample of volunteers of all ages, they found that divorced volunteers especially had to reduce their volunteering time.

The most important policy message delivered by the results of this study is that they seem to provide empirical support to the thesis of the inappropriateness of the ageist approach adopted by governments during the COVID-19 pandemic. For social and (to some extent) altruistic reasons, older volunteers may aspire to continue to serve as volunteers and to increase volunteering time in emergency situations [59]. Policy makers should consider that in the face of possible future emergency situations of different types (e.g., health crisis, earthquakes, floods, etc.), older people should not be treated as a homogeneous group [19], thus, governments should avoid implementing policies based on “the tyranny of averages” [11], since the latter is an approach that neglects diversity. This view is strengthened by the results of this study, which highlight that decisions regarding older people’s voluntary service are not affected by the fact that they may have experienced, directly or (closely) indirectly, COVID-19 symptoms. The fact that the present study was conducted in the context of the COVID-19 pandemic has clearly affected all the results obtained. However, the latter result could indicate that lessons from this study about the volunteering habits of older people could be learned and useful not just during a time of emergency. The amount of older people in society is increasing; they are supposed to have enough time to devote to volunteer activities and they seem to be especially driven by social-related volunteer motivations. This may be important information for voluntary organizations dealing with the need to recruit volunteers through recruitment campaigns.

This study has some limitations. A main limitation is that it is an explorative study, conducted on a sample that is not representative of the whole population of older volunteers; therefore, the results obtained cannot be generalized for the wider Italian context. Furthermore, Italy was the Western country most affected by the COVID-19 outbreak in the first pandemic wave, and it was characterized by stricter containment measures compared to other countries, thus leading to the need to develop future research on this topic in other countries, preferably from a cross-country perspective. Moreover, given that the present study deals with older volunteers, future research should explore whether these findings would also apply to younger generations by studying both younger and older volunteers. Furthermore, since the study was intended to explore possible changes in the volunteering habits of active volunteers, it does not capture the experiences of older volunteers who quit volunteering during the pandemic, a phenomenon that may have been of particular concern for older volunteers [60]. It is not excluded that among those older volunteers reporting changes in volunteer activities and/or a reduction of volunteering time, there are some cases of volunteers who quit volunteer work. Despite these limitations, this study adds to the literature by providing original results regarding the volunteer experience of older people during the pandemic by exploring possible changes in their volunteering habits and the reasons for those changes.

## 5. Conclusions

This study found that during the COVID-19 pandemic, older volunteers especially driven by social goals (e.g., opportunities to have relationships with others) were able to continue volunteer activities without any need for change. This may be due to the fact that through volunteering, these individuals tried to counter the undesired calls from the government for self-isolation and physical distancing from other individuals. Having direct experience with COVID-19 symptoms did not seem to deter older volunteers in this respect, as it was possible to affirm this after having controlled for this aspect through the analyses. As an additional result, it was shown that weak, older individuals driven by altruistic reasons may have desired to increase their volunteering time during the pandemic. These results have important policy implications, making clear that in the event of future emergency situations involving older people, policy makers should not treat them only as recipients of help and care, but also as providers of help in the community [61], according to their health status, possibilities, intentions and motivations.

## Figures and Tables

**Table 1:** Sample description (N = 240).

Variables	%; Means (sd)
*Demographics and socio-economic characteristics*	
Age	70.26 (6.4)
Gender (female)	47.9
Marital status:	
Married/cohabiting	71.6
Single, separated or divorced	16.3
Widowed	12.1
Educational level:	
Low	38.3
Intermediate	44.2
High	17.5
*Volunteering motivational functions*	
Values	3.86 (0.7)
Understanding	3.33 (0.9)
Enhancement	3.45 (0.9)
Social	3.09 (0.9)
Protective	2.77 (1.0)
Career	1.86 (1.0)
*COVID-19-related factors*	
I have had COVID-19	13.0
A family member has had COVID-19	28.0
An acquaintance has had COVID-19	46.5
*Volunteering-related outcome variables*	
I continued to carry out the same activities (yes)	49.8
I changed the volunteering activities (yes)	61.1
I increased volunteering time (yes)	23.1
I decreased volunteering time (yes)	41.7

**Table 2:** A. Possible changes in volunteering activities and time and motivational functions, bivariate analyses, means. B. Possible changes in volunteering activities and time and motivational functions, bivariate analyses, means.

**A**						
	**Values**		**Understanding**		**Enhancement**	
	**Mean (sd)**	***p***	**Mean (sd)**	***p***	**Mean (sd)**	***p***
**Same volunteering activities**						
Yes	3.94 (0.7)	0.453	3.46 (0.9)	0.093	3.55 (0.9)	0.225
No	3.86 (0.7)		3.25 (0.9)		3.40 (0.9)	
**Changed volunteering activities**						
Yes	3.94 (0.7)	0.311	3.33 (0.9)	0.638	3.46 (0.9)	0.781
No	3.83 (0.8)		3.39 (0.9)		3.50 (0.9)	
**Increased volunteering time**						
Yes	4.20 (0.7)	0.001 ***	3.73 (0.8)	0.001 ***	3.71 (0.9)	0.032 *
No	3.81 (0.7)		3.24 (0.9)		3.40 (0.9)	
**Decreased volunteering time**						
Yes	3.81 (0.7)	0.154	3.23 (0.9)	0.132	3.35 (0.9)	0.116
No	3.95 (0.7)		3.42 (0.9)		3.55 (0.9)	
**B**						
	**Social**		**Protective**		**Career**	
	**Mean (sd)**	***p***	**Mean (sd)**	***p***	**Mean (sd)**	***p***
**Same volunteering activities**						
Yes	3.31 (0.9)	0.001 ***	2.93 (0.9)	0.028 *	2.02 (1.0)	0.012 *
No	2.88 (0.9)		2.63 (1.0)		1.68 (0.9)	
**Changed volunteering activities**						
Yes	2.99 (1.0)	0.064	2.75 (1.0)	0.597	1.83 (1.0)	0.748
No	3.24 (0.9)		2.82 (1.0)		1.88 (1.0)	
**Increased volunteering time**						
Yes	3.49 (0.8)	0.001 ***	3.05 (1.0)	0.018 **	2.08 (1.0)	0.048 *
No	2.96 (0.9)		2.66 (1.0)		1.75 (0.9)	
**Decreased volunteering time**						
Yes	2.83 (1.0)	0.001 ***	2.56 (1.0)	0.018 **	1.63 (0.9)	0.015 *
No	3.26 (0.9)		2.89 (1.0)		1.97 (1.0)	

* *p* < 0.05; ** *p* < 0.01; *** *p* ≤ 0.001.

**Table 3:** Possible changes in volunteering activities and time and experience with COVID-19 (yes), bivariate analyses, %.

	COVID-19 Myself		COVID-19 Family Member		COVID-19 Acquaintance	
	%	*p*	%	*p*	%	*p*
**Same volunteering activities**						
Yes	13.0	0.979	31.5	0.258	54.6	0.011 *
No	12.8		23.9		36.7	
**Changed volunteering activities**						
Yes	11.4	0.373	26.5	0.622	41.7	0.107
No	15.5		28.6		52.4	
**Increased volunteering time**						
Yes	12.2	0.884	24.5	0.687	51.0	0.274
No	12.9		27.6		42.3	
**Decreased volunteering time**						
Yes	9.1	0.192	21.6	0.175	37.5	0.112
No	15.4		30.9		49.6	

* *p* < 0.05.

**Table 4:** Explanatory variables for continuing to carry out the same volunteering activities in the time of COVID-19 (yes), logistic regression.

	MODEL 1		MODEL 2		MODEL 3		MODEL 4	
	OR	*p*	OR	*p*	OR	*p*	OR	*p*
**Motivational functions**								
Values	0.97	0.926			0.94	0.839	1.00	0.991
Understanding	0.94	0.858			0.98	0.961	0.60	0.178
Enhancement	0.69	0.220			0.69	0.231	0.86	0.698
Social	1.74	0.017 *			1.88	0.009 **	2.43	0.003 **
Protective	1.09	0.701			1.04	0.854	1.15	0.632
Career	1.29	0.211			1.28	0.241	1.20	0.460
**Experience of COVID-19**								
I have had COVID-19			0.87	0.752	0.811	0.658	0.76	0.643
A family member has had COVID-19			1.36	0.354	1.07	0.848	1.18	0.705
An acquaintance has had COVID-19			2.11	0.009 **	2.03	0.024 *	1.30	0.487
**Control variables**								
Age							0.94	0.101
*Gender* (*ref. Male*)								
Female							0.72	0.456
*Marital status* (*ref. Married/cohabiting*)								
Single/separated/divorced							1.68	0.376
Widowed							1.15	0.811
*Educational level* (*ref. Low*)								
Intermediate							1.37	0.460
high							1.54	0.446
*Volunteering frequency* (*ref. Twice a week or more often*)								
Once a week							0.25	0.002 **
Once every two weeks							0.22	0.014 *
Once a month or less often							0.04	0.001 ***
*Informal family elder care* (*ref. No*)								
Yes							3.46	0.055

* *p* < 0.05; ** *p* < 0.01; *** *p* ≤ 0.001.

**Table 5:** Explanatory variables for changing volunteering activities in times of COVID-19 (yes), logistic regression.

	MODEL 1		MODEL 2		MODEL 3		MODEL 4	
	OR	*p*	OR	*p*	OR	*p*	OR	*p*
**Motivational functions**								
Values	1.55	0.134			1.52	0.155	1.47	0.291
Understanding	0.83	0.562			0.85	0.612	0.88	0.730
Enhancement	1.38	0.269			1.40	0.260	1.65	0.153
Social	0.58	0.027 *			0.62	0.052	0.59	0.066
Protective	1.00	0.994			0.97	0.928	0.75	0.343
Career	1.05	0.789			1.05	0.801	1.13	0.626
**Experience of COVID-19**								
I have had COVID-19			0.67	0.356	0.83	0.699	0.57	0.326
A family member has had COVID-19			0.97	0.935	1.00	0.979	0.94	0.898
An acquaintance has had COVID-19			0.64	0.135	0.67	0.217	0.64	0.258
**Control variables**								
Age							1.04	0.225
*Gender* (*ref. Male*)								
Female							1.71	0.196
*Marital status* (*ref. Married/cohabiting*)								
Single/separated/divorced							0.82	0.711
Widowed							4.6	0.038 *
*Educational level* (*ref. Low*)								
Intermediate							0.50	0.108
high							1.15	0.808
*Volunteering frequency* (*ref. Twice a week or more often*)								
Once a week							3.05	0.015 *
Once every two weeks							4.79	0.018 *
Once a month or less often							0.39	0.181
*Informal family elder care* (*ref. No*)								
Yes							2.59	0.101

* *p* < 0.05.

**Table 6:** Explanatory variables for increasing volunteering time in the time of COVID-19 (yes), logistic regression.

	MODEL 1		MODEL 2		MODEL 3		MODEL 4	
	OR	*p*	OR	*p*	OR	*p*	OR	*p*
**Motivational functions**								
Values	2.22	0.032 *			2.34	0.024 *	2.25	0.055
Understanding	1.36	0.387			1.32	0.438	1.05	0.885
Enhancement	0.88	0.708			0.92	0.831	0.81	0.620
Social	1.29	0.342			1.33	0.292	1.46	0.212
Protective	0.78	0.400			0.83	0.546	1.06	0.853
Career	1.15	0.526			1.02	0.907	1.11	0.681
**Experience of COVID-19**								
I have had COVID-19			1.03	0.952	1.16	0.780	1.40	0.585
A family member has had COVID-19			0.84	0.665	0.59	0.251	0.75	0.588
An acquaintance has had COVID-19			1.44	0.275	1.54	0.253	1.34	0.490
**Control variables**								
Age							1.00	0.908
*Gender* (*ref. Male*)								
Female							1.33	0.534
*Marital status* (*ref. Married/cohabiting*)								
Single/separated/divorced							1.10	0.871
Widowed							0.20	0.065
*Educational level* (*ref. Low*)								
Intermediate							1.51	0.373
high							0.83	0.789
*Volunteering frequency* (*ref. Twice a week or more often*)								
Once a week							0.45	0.119
Once every two weeks							0.17	0.046 *
Once a month or less often							0.28	0.184
*Informal family elder care* (*ref. No*)								
Yes							0.87	0.827

* *p* < 0.05.

**Table 7:** Explanatory variables for decreasing volunteering time in the time of COVID-19 (yes), logistic regression.

	MODEL 1		MODEL 2		MODEL 3		MODEL 4	
	OR	*p*	OR	*p*	OR	*p*	OR	*p*
**Motivational functions**								
Values	0.77	0.361			0.80	0.457	1.00	0.992
Understanding	1.36	0.317			1.38	0.299	1.69	0.165
Enhancement	1.06	0.818			1.01	0.972	0.93	0.854
Social	0.65	0.064			0.60	0.038 *	0.57	0.052
Protective	0.91	0.710			0.96	0.896	0.66	0.179
Career	0.82	0.364			0.82	0.401	0.91	0.717
**Experience of COVID-19**								
I have had COVID-19			0.63	0.333	0.56	0.266	0.28	0.052
A family member has had COVID-19			0.73	0.372	0.93	0.862	0.90	0.830
An acquaintance has had COVID-19			0.58	0.075	0.61	0.137	0.62	0.230
**Control variables**								
Age							1.06	0.102
*Gender* (*ref. Male*)								
Female							1.21	0.634
*Marital status* (*ref. Married/cohabiting*)								
Single/separated/divorced							0.74	0.594
Widowed							6.53	0.004 **
*Educational level* (*ref. Low*)								
Intermediate							0.76	0.539
high							0.62	0.413
*Volunteering frequency* (*ref. Twice a week or more often*)								
Once a week							2.15	0.079
Once every two weeks							3.25	0.059
Once a month or less often							5.41	0.022 **
*Informal family elder care* (*ref. No*)								
Yes							1.33	0.621

* *p* < 0.05; ** *p* < 0.01.

## Data Availability

Data are available upon request to the authors.
